# Global landscape of 2-hydroxyisobutyrylation in human pancreatic cancer

**DOI:** 10.3389/fonc.2022.1001807

**Published:** 2022-09-30

**Authors:** Yun Lu, Xiangyu Li, Kai Zhao, Peng Qiu, Zhengdong Deng, Wei Yao, Jianming Wang

**Affiliations:** ^1^ Department of Biliary and Pancreatic Surgery, Cancer Research Center Affiliated Tongji Hospital, Tongji Medical College, Huazhong University of Science and Technology, Wuhan, China; ^2^ Department of Oncology Affiliated Tongji Hospital, Tongji Medical College, Huazhong University of Science and Technology, Wuhan, China; ^3^ Affiliated Tianyou Hospital, Wuhan University of Science & Technology, Wuhan, China

**Keywords:** PTMs, post-translational modifications, Khib, lysine 2-hydroxyisobutyrylation, pancreatic cancer, proteomics

## Abstract

As a new type of post-translational modification (PTM), lysine 2-hydroxyisobutyrylation (K_hib_) was firstly identified in histones and functioned as a regulator of transactivation in mammals. However, the role of K_hib_ proteins remains to be investigated. Here, we firstly identified 10,367 K_hib_ sites on 2,325 modified proteins in seven patients with pancreatic cancer by applying liquid chromatography with tandem mass spectrometry (LC-MS/MS) qualitative proteomics techniques. Among them, 27 K_hib_-modified sites were identified in histones. Bioinformatics analysis revealed that the K_hib_-modified proteins were mainly distributed in the cytoplasm and enhanced in metabolic pathways, including glycolysis/gluconeogenesis, the tricarboxylic acid cycle (TCA cycle), and fatty acid degradation. In an overlapping comparison of lysine 2-hydroxyisobutyrylation, succinylation, and acetylation in humans, 105 proteins with 80 sites were modified by all three PTMs, suggesting there may be a complex network among the different modified proteins and sites. Furthermore, MG149, which was identified as a Tip60 inhibitor, significantly decreased the total Khib modification level in pancreatic cancer (PC) and strongly suppressed PC’s proliferation, migration, and invasion ability. Overall, our study is the first profiling of lysine 2-hydroxyisobutyrylome and provides a new database for better investigating K_hib_ in PC.

## Highlights

1. This is the first report of K_hib_ in the tumor tissue of patients with pancreatic cancer.
2. The K_hib_ proteins in pancreatic cancer are widely distributed.
3. Inhibiting the total Khib level can suppress pancreatic cancer cells’ proliferation, migration, and invasion ability.

## Introduction

Post-translational modifications (PTM) of proteins contribute to protein function by adding chemical groups to amino acid residues to alter the charge, structure, and molecular weight of the protein (1), thereby affecting protein activity, subcellular location, interaction chaperones, stability, and amplifying/weakening protein function (2). Until now, more than 200 PTMs have been identified, including phosphorylation, ubiquitination, acetylation, succinylation, and 2-hydroxyisobutyrylation (3–5).

It is universally acknowledged that 2-hydroxybutyrate (2-HIBA) is a microbial metabolite detected at micromolar concentrations in human bio-fluids, including blood, saliva, and urine (6). Previous reports demonstrated that elevated 2-HIBA levels were associated with metabolic diseases, including diabetes (7) and obesity (8). 2-hydroxyisobutyrate was a synthetic precursor of 2-hydroxyisobutyryl coenzyme A, which was regulated by carbon sources (9) and identified as the donor of lysine 2-hydroxyisobutyrylation (K_hib_) (10). Lysine 2-hydroxyisobutyrylation is a novel PTM, which was first detected in histones in 2014 (4) and has been validated by following reports in animals, plants, and microorganisms (11–14). Tip60 was identified as a K_hib_ writer in mammalian cells, which could catalyze K_hib_ modifications *in vitro* and *in vivo*. In contrast, histone deacetylase 2 (HDAC2) and histone deacetylase 3 (HDAC3), but not histone deacetylase 1 (HDAC1), acted as erasers to remove K_hib_ modifications *in vivo (* 14). Previous studies have confirmed that K_hib_ is a widespread type of PTM with strict evolutionary conservation, and it could be up-regulated by carbon sources generated by the pentose phosphate pathway and glycolysis/gluconeogenesis (12), which strongly suggestes a close correlation between K_hib_ modification and metabolism. Recently, K_hib_ was reported to be involved in cancer progression. K_hib_ was identified in ENO1 and promoted the ability of glycolysis and proliferation in hepatoma cells (15). Also, K_hib_ in oral squamous cell carcinoma (OSCC) was suggested to promote cancer progression (16).

Pancreatic cancer (PC) is one of the most malignant digestive tumors, with a five-year survival rate of only 9%. In the past decade, the mortality rate of men with PC has increased steadily, rather than decreasing. According to a statistical report, there were up to 420,000 new PC cases and 410,000 deaths worldwide in 2020 (17), and it is predicted that PC may become the second most deadly malignancy in 2030, just after lung cancer (18). Currently, the treatment of PC is mainly surgical resection described as pancreaticoduodenectomy; however, owing to its insidious onset, high malignancy, and rapid progression, the operable rate is far below 20% (19). Post-translational modifications have a vital role in epigenetics and signal transduction, numbers of studies have focused on the contribution of PTMs as diagnostic markers or therapeutic targets for cancer treatment (20). Although there have been many reports on lysine 2-hydroxyisobutyrylation in recent years, the role of K_hib_ in PC development remained unclear.

In the present study, we first reported the K_hib_ sites in human pancreatic cancer *via* proteomics. Totally, 10,367 lysine 2-hydroxyisobutyrylation modification sites on 2325 proteins were identified, and they mainly enriched in protein synthesis and metabolic pathways. Moreover, MG149, a selective inhibitor of Tip60, was proved to enormously decrease the total Khib level in PC and suppress PC cell proliferation and metastasis abilities. The novel K_hib_ sites and proteins identified in this research not only promote our insights into the functional role of K_hib_ but also provide new perspectives and potential drug targets for future investigations in PC.

## Result

### The lysine 2-hydroxyisobutyrylation modification in pancreatic cancer

**Figure 1:**
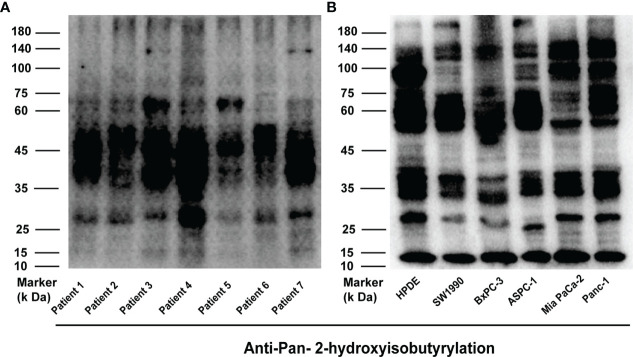
2-hydroxyisobutyrylation (K_hib_) is a type of widely distributed protein-translational modifications (PTMs). **(A, B)** Western blot analysis of the Khib proteins in pancreatic cancer (PC) and every cell line.

To demonstrate the K_hib_ modification stage in PC, the tissues from seven different patients (**Table S1**) were digested and lysed to extract proteins (**Figure S1A**), and then Western Blotting with pan- 2-hydroxyisobutyryllysine antibody (pan-anti-K_hib_) was used to prove the status of K_hib_ in these proteins (**Figure 1A**). Also, proteins obtained from different pancreatic cell lines (HPDE, SW1990, BXPC, ASPC, Mia PaCa-2, PANC-1) were examined by WB (**Figure 1B** and **Figure S1B**). Our analysis showed that the level of K_hib_ modification in different PC tissues or cell lines was slightly different, mainly concentrated at 35—75 kD, indicating Khib was widely distributed and conserved in PC.

### Systematic analysis of lysine 2-hydroxyisobutyrylation in pancreatic cancer

**Figure 2:**
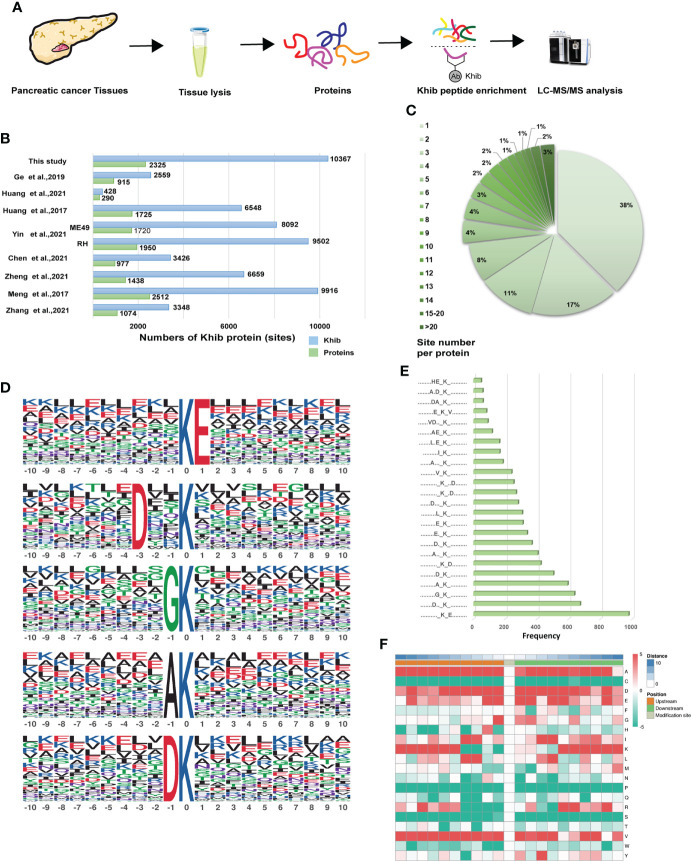
Genome-wide identity and profiling of the K_hib_ proteins and sites in PC. **(A)** Workflow for the identification of K_hib_ modification in PC. Proteins extracted from the PC tissues were digested into peptides, and then the specific K_hib_ antibody enriched the K_hib_ sites-containing peptides, followed by the liquid chromatography with tandem mass spectrometry (LC-MS/MS) analysis. **(B)** Comparison of the K_hib_ proteins and sites identified in our study and those in previous reports. **(C)** Numbers and ratios of K_hib_ sites for each protein. **(D)** The top five K_hib_ motifs. The size of each letter indicates the frequency of that amino acid residue at that position. **(E)** Frequency of occurrence of each motif among the peptides with K_hib_ modifications. **(F)** The heatmap of the amino acid profile of the K_hib_ sites, featuring the enrichment (red) and depletion (green) of the amino acids at every position (from -10 to +10) on both sides of the K_hib_ site.

Based on the antibody enrichment and liquid chromatography with tandem mass spectrometry (LC-MS/MS) methods, proteomic analysis was performed on seven pancreatic cancer tissue samples extracted from patients with PC (**Figure 2A**). In this study, a total of 10,367 K_hib_-modified sites in 2,325 proteins were identified with a localization probability > 0.75. The details of all proteins and associated K_hib_ sites identified in this study were listed in Table S2. Our study showed a great depth of detection in K_hib_ proteins compared to previous studies and contributed to a more comprehensive K_hib_ map in PC (**Figure 2B**). To characterize the extent of K_hib_ coverage in the substrate proteins, we calculated the number of every protein modification site. Most proteins showed 1–3 modification sites, 38%, 17%, and 11%, respectively. In comparison, the percentage of proteins with > 20 sites was 3% (**Figure 2C**), demonstrating that K_hib_ modifications always have more than one site in most of the proteins, suggesting us to pay attention to the position of modification and possible interrelationships in the subsequent protein modification studies.

The Motif-X program was performed to detect the sequence motifs in the K_hib_-modified peptides (**Table S3**). The five most abundant motifs were [K_hib_E], [DXXK_hib_], [GK_hib_], [AK_hib_] and [DK_hib_] (in which X indicates random amino acid residue) with frequency of 978, 672, 636, 594, and 504 respectively (**Figures 2D, E**). The motif enrichment heatmap showed a significant preference for negatively charged glutamate (E) and aspartate (D) around the modified lysine residue. In contrast to previous studies, alanine (A) and valine (V) showed great preference around the modified lysine residue (**Figure 2F**), which indicated the complex nature of the potential PTM mechanism in PC.

### Functional analysis of the lysine 2-hydroxyisobutyrylated proteins in PC

**Figure 3:**
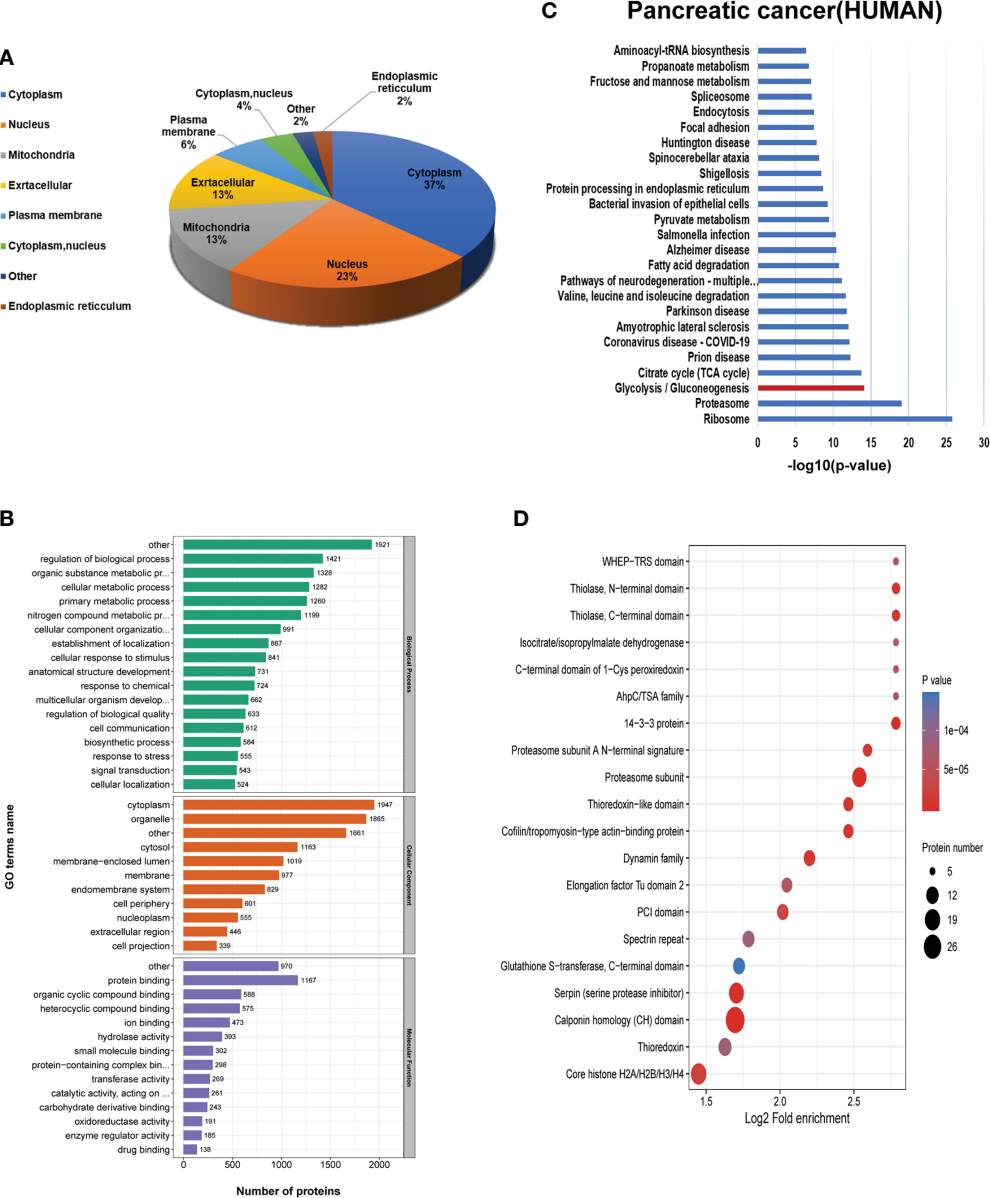
Bioinformatics analysis of the 2-hydroxyisobutyrylation (K_hib_) proteins. **(A)** Subcellular localization of the K_hib_ proteins. **(B)** Gene Ontology (GO) classification analysis of the K_hib_ proteins based on the biological process, molecular function, and cellular component. **(C)** Kyoto Encyclopedia of Genes and Genomes (KEGG) pathway analysis of the K_hib_ proteins. **(D)** Protein domain enrichment analysis.

To systematically describe the K_hib_ modified proteins, Wolfpsort software was applied to analyze the subcellular location of the 2-hydroxyisobutyrylated proteins in PC. There were 37%, 23%, 13%, 13%, and 6% K_hib_ proteins distributed in the cytoplasm, nucleus, mitochondria, extracellular, and plasma membrane, respectively (**Figure 3A**), suggesting that the K_hib_ modification was widespread in PC. Then, Gene Ontology (GO) analysis, including biological processes, cellular components, and molecular functions, was applied to demonstrate the potential functions and locations of the K_hib_ proteins (**Table S4**). According to the cellular component data, K_hib_ proteins were enriched in the cytoplasm (27%), organelles (16%), membrane-enclosed lumen (9%), and membrane (9%). In accordance with the molecular function analyses, the K_hib_ proteins were mainly associated with binding (63%) and enzyme activity (21%), revealing that K_hib_ modification may play a key role in protein function regulation, DNA transcription, and metabolic activity. The biological process analyses presented that the K_hib_ proteins were mainly distributed in the metabolism (31%), biological process (13%), stimulation response (12%), and location (7%) (**Figure 3B** and **Figure S3A**).

Furthermore, to explore the characteristics and role of the K_hib_ proteins in PC, Kyoto Encyclopedia of Genes and Genomes (KEGG) pathway analysis was performed to show that proteins with K_hib_ modification concentrated in metabolic pathways, including glycolysis/gluconeogenesis, TCA cycle, and fatty acid degradation, among others, in accordance with a previous study on *Oryza sativa (* 11) and *Ustilaginoidea virens (* 21) (**Figure S3C** **).** Also, ribosomes, proteasomes, and proteins related to multiple diseases were enriched in the KEGG pathways analysis, indicating that the K_hib_ proteins had a close interrelationship with protein synthesis, degradation, bio-metabolism, and disease development (**Figures 3C, B**). The K_hib_ proteins were observed intensively in the proteasome subunit, 14-3-3 protein, serpin (serine protease inhibitor), cofilin/tropomyosin-type actin-binding protein, thioredoxin-like domain, and Core histone H2A/H2B/H3/H4 in the protein domain enrichment analysis (**Figure 3D**).

### The conservation and specificity of the lysine 2-hydroxyisobutyrylated proteins

**Figure 4:**
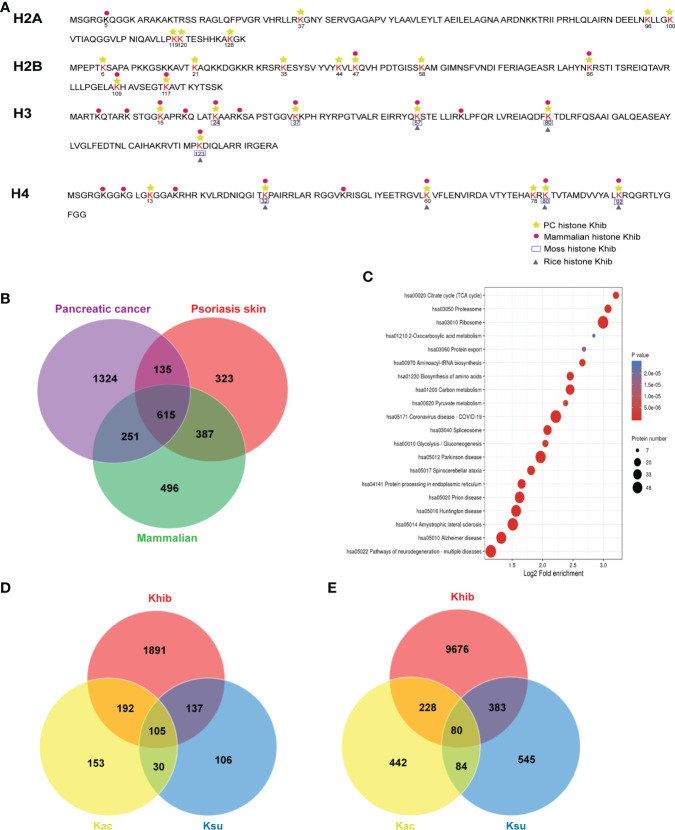
Conservation and uniqueness of the 2-hydroxyisobutyrylation (K_hib_) proteins in human pancreatic cancer (PC). **(A)** The comparison of the K_hib_ histones in diverse species. **(B)** The number of overlapping K_hib_ proteins among PC, psoriasis skin, and mammalian cells. **(C)** Kyoto Encyclopedia of Genes and Genomes **(KEGG)** analysis of the common K_hib_ proteins in PC, psoriasis skin, and mammalian cells. **(D)** The number of overlapping proteins among K_hib_, lysine succinylation (K_su_), and lysine acetylation (K_ac_). **(E)** The number of overlapping modified sites among K_hib_, K_ac_, and K_su_.

Lysine 2-hydroxyisobutyrylation was first reported in the histones and was critical for regulating chromatin function (4). To investigate the K_hib_ modification status of histones in PC, we listed 27 K_hib_ sites on the histones identified in this study (**Table S5**), and a comparison was performed with other species, including rice seed (22), mammals (14), and moss (23) (**Figure 4A**), which revealed multiple overlapping K_hib_ modification sites on the H3 and H4 proteins, and the specific sites H4K13 and H4K78 were identified. Among these, nine sites on H2B were observed, which were mainly distributed between K34-K120 in the globular structure, and it may affect the binding of H2B to DNA by shifting the charge state (4). Overall, different species have their unique K_hib_ sites, suggesting that K_hib_ is not only conserved but is also a unique PTM in histones.

Simultaneously, the K_hib_ modification in proteins excluding histones showed its features. By comparing the K_hib_ proteins identified in mammalian (HeLa cells) (14), psoriasis skin (24), and PC detected in this study, 615 Khib proteins were observed in all the reports (**Figure 4B** and **Table S6**). The KEGG enrichment analysis of these common K_hib_ proteins showed a preferred distribution in the ribosome and metabolism, including glycolysis/gluconeogenesis, carbon metabolism, and TCA cycle (**Figure 4C**), indicating that K_hib_ modification was conserved among different species. Meanwhile, the K_hib_ proteins, especially those populated in PC, were enriched in the metabolic, phosphatidylinositol 3-kinase (PI3K)-protein kinase B (Akt) signaling, and tight junction pathways (**Table S6** and **Figure S6**), indicating that K_hib_ modification was of great significance in the fields of bio-metabolism in PC

### Overlap of lysine 2-hydroxyisobutyrylation and other PTMs

Post-translational modifications played crucial roles in regulating protein functions. It has been reported that various types of PTMs can be observed in one protein or even in the same locus. Compared with the results of previous studies in humans (25, 26), a total of 105 proteins with 80 sites were identified to be modified by K_hib_, lysine succinylation (K_su_), and lysine acetylation (K_ac_) (**Figure 4D** and **Table S6**). In addition, 242 and 297 K_hib_ proteins presented K_su_ and K_ac_ modifications, respectively. In addition, 463 and 308 K_hib_ sites were shared with K_su_ and K_ac_, respectively (**Figure 4E** and **Table S6**). Among them, some lysine sites closely related to protein activity showed multiple modifications, suggesting that K_hib_ might be biologically meaningful.

### Lysine 2-hydroxyisobutyrylation in central metabolism

**Figure 5:**
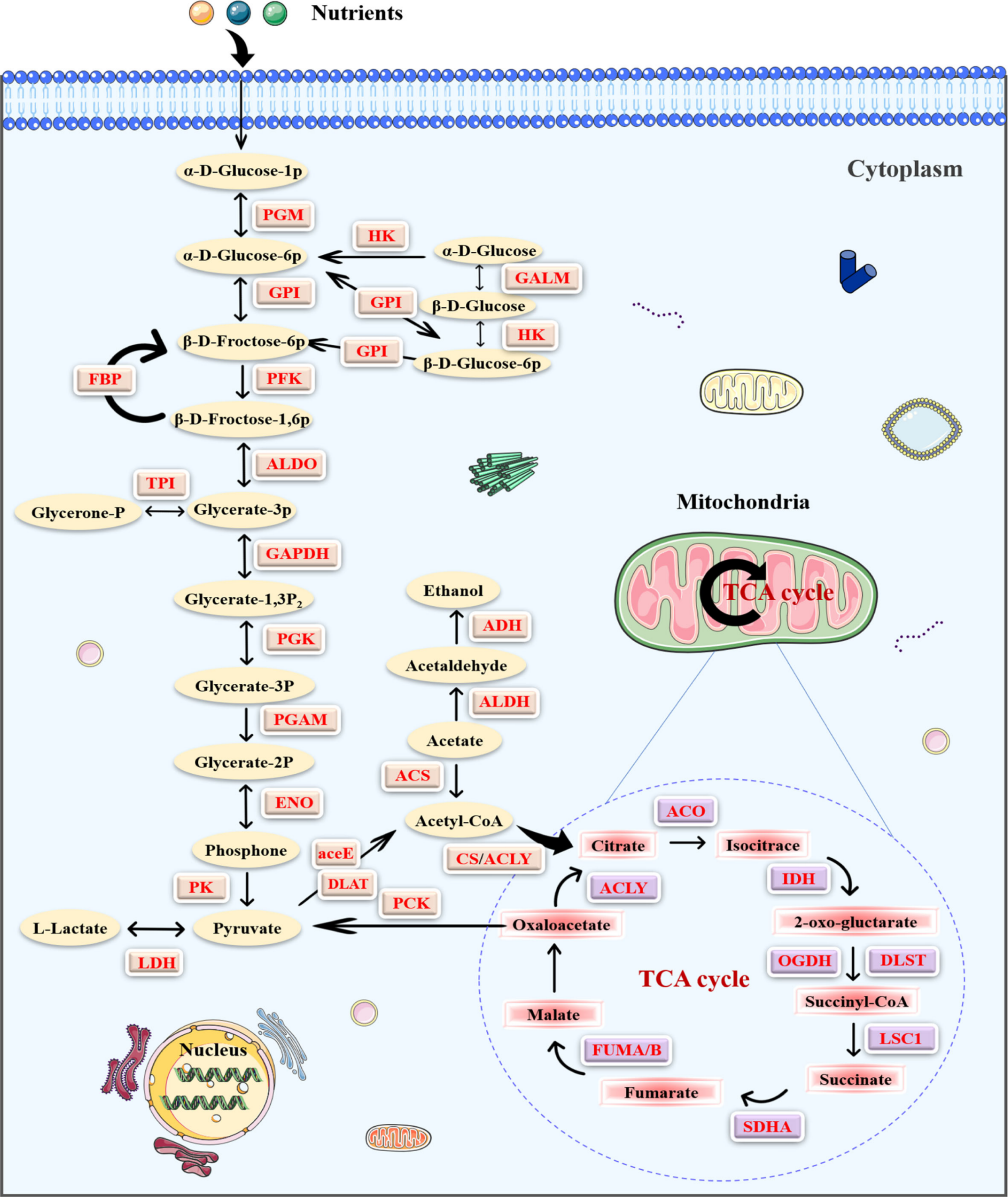
The enrichment of the 2-hydroxyisobutyrylation (K_hib_) proteins in the central metabolic pathways in pancreatic cancer (PC). The K_hib_ enzymes are marked with red.

As described in the KEGG analysis, K_hib_ proteins were concentrated in metabolic pathways in PC (**Figure 3C**). Compared with other species (**Figure S3C** **)** (11, 21), glycolysis/gluconeogenesis was observed in the K_hib_ proteins analysis, suggesting that K_hib_ plays a critical role in regulating glucose metabolism. Further, many enzymes in central metabolism, including glycolysis/gluconeogenesis, TCA cycle, and pentose phosphate metabolic pathways, were mapped to study the function of K_hib_ in PC (**Figure 5**). It is worth noting that some critical enzymes represented multiple modification sites, including PGK, glyceraldehyde 3-phosphate dehydrogenase (GAPDH), and lactate dehydrogenase (LDH) (**Table S7**). Clearly, K_hib_ modifications were prevalent in metabolic enzymes, and the modification status of specific sites affected enzyme activity and served a vital role in various metabolic pathways.

### Inhibiting lysine 2-hydroxyisobutyrylation level suppressed PC proliferation, migration, and invasion

**Figure 6:**
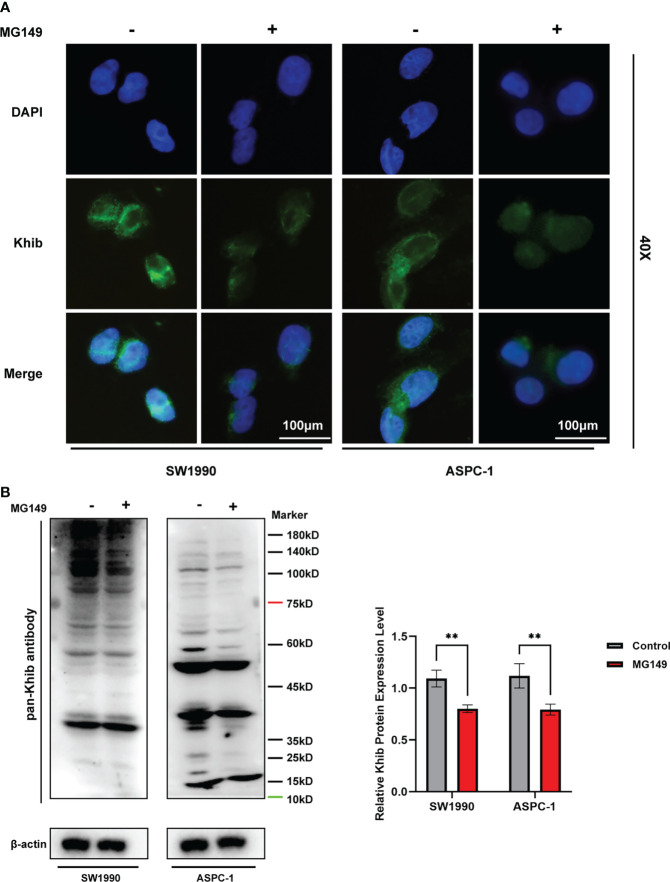
The total 2-hydroxyisobutyrylation (K_hib_) level in pancreatic cancer (PC) cells were suppressed by MG149. The SW1990 and ASPC-1 cells were treated with MG149 (74 μM), and then the immunofluorescence and Western blot assays were performed with pan-K_hib_ antibody. **(A)** Immunofluorescence assay for PC cells treated with MG149. Different stains represent specific target genes, K_hib_ (green) and DAPI (blue). Scale bar, 100 μm. **(B)** Western blotting analysis of the K_hib_ proteins in the SW1990 and ASPC-1 cells treated with MG149. **P < 0.01, determined by independent Student’s t tests. Data are derived from three independent experiments and presented as means ± SDs.

**Figure 7:**
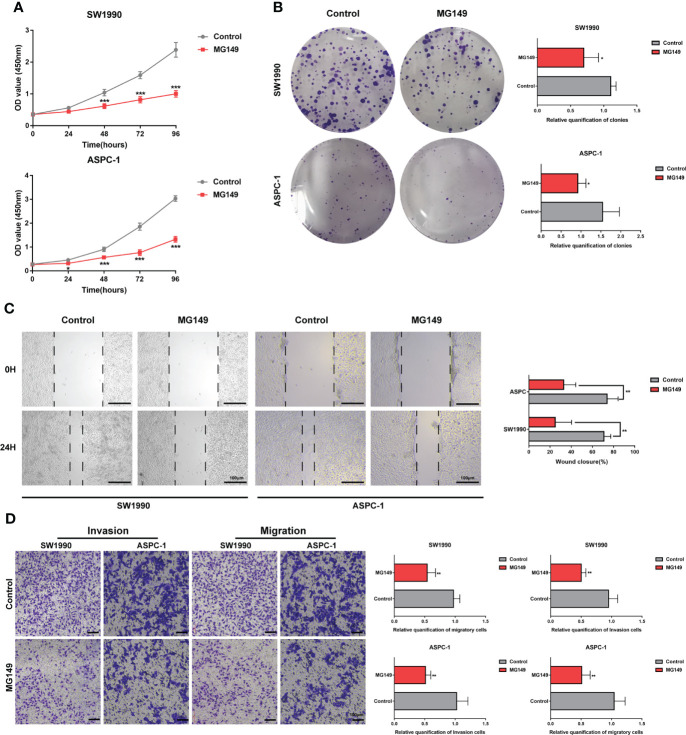
MG149 suppressed PC proliferation, migration, and invasion. **(A)** CCK8 assay was performed to measure the proliferation ability of PC cells. **(B)** Representative images of the colony formation assay of the SW1990 and ASPC-1 cells with or without MG149 (74 μM) treatment and culture in a complete culture medium for 14 days. **(C)** Representative images of the wound healing assays of the SW1990 and ASPC-1 cells with or without MG149 (74 μM) treatment, Scale bar: 100 μm. **(D)** Transwell assays were performed to examine the effect of MG149 on PC cell migratory and invasion ability. Scale bar: 100 μm. *P < 0.05, **P < 0.01, ***P < 0.001.

MG149 was identified as a selective inhibitor of Tip60 (27), which was known as a “writer” of K_hib_ modification in mammalian cells (14). When treated with MG149, the level of K_hib_ modification in PC cells was significantly decreased (**Figures 6A, B** and **Figure S5**). In order to investigate the role of K_hib_ in PC development, the CCK8, colony-forming, wounding, and transwell assays were performed with the SW1990 and ASPC-1 cells. The CCK8 and colony-forming assay results suggested that MG149 contributed to reduced viability in the SW1990 and ASPC-1 cells (**Figures 7A, B**). Besides, the results of wounding and transwell assay demonstrated that the ability of migration and invasion was significantly suppressed when PC cells were treated with MG149 (**Figures 7C, D**). Thus, the results above strongly suggested that lysine 2-hydroxyisobutyrylation affected the PC cells’ proliferation, migration, and invasion ability *in vitro*.

## Discussion

Post-translational modifications of proteins, including phosphorylation, acetylation, and lactylation, alter protein function by adding chemical groups. 2-hydroxyisobutyrylation is a novel type of PTM identified in histones, and it played a crucial role in the regulatory function of chromatin (4) with operational differences from acetylation modifications (28). K_hib_ is prevalent in several species, including plants, microorganisms, and humans. Recently, studies on K_hib_ have been extended to non-histone proteins, and it is suggested to be involved in the development of human diseases. The K_hib_ proteins identified in peripheral blood from patients with immunoglobulin A (IgA) nephropathy aggregate in the interleukin-17 (IL-17) signaling pathway and phagosome class (29), and they were also observed to be enriched in the PI3K-Akt signaling pathway in psoriasis skin lesions, which is associated to psoriasis progression (24). Conversely, K_hib_ has rarely been studied in pancreatic cancer. To investigate the role of K_hib_ in the progression of PC, we identified 10367 Khib sites on 2325 proteins in this study, which were more compared to that in previous studies, and expanded the databases of K_hib_ proteins in PC.

In agreement with previous studies, 27 K_hib_ sites in histones were identified in this study, of which the modification sites in H2B were mainly concentrated in the spherical structure of K34-K120, which may have affected the binding of H2B to DNA (4). Given that there are fewer studies on K_hib_ in humans, for reasons of homology, we chose Khib data from mammalian cells and psoriasis skin in humans to perform a comparison with K_hib_ proteins in PC. Notably, 615 K_hib_ proteins were found in the above studies, and they were predicted to make great contributions to regulating metabolism pathways, including the TCA cycle, glycolysis, and carbon metabolism. In addition, 105 proteins and 80 sites were found to show three modifications by overlapping analysis with acetylation, succinylation, and 2-hydroxyisobutyrylation. This may result from regulation by the transcriptional co-activator P300, which was suggested to catalyze various modifications of histones, including acetylation, propionylation, butyrylation, and 2-hydroxyisobutyrylation (6, 28, 30, 31). Therefore, it is reasonable to suppose that there is either synergistic or competitive crosstalk among different types of PTMs, suggesting the possibilities of multiple modifications of proteins should be fully considered in studies of proteins, highlighting the importance and necessity of protein modification mapping.

Pancreatic tumor is exposed to severe physical, oxidative, and inflammatory stress. Thus, cancer cells rewire intermediary metabolism to promote unlimited proliferation in a hypoxic-ischemic environment, evade immune surveillance, and develope distant metastases. Previous studies have demonstrated that metabolic reprogramming promotes pancreatic tumorigenesis and metastasis through epigenetic regulation (32, 33). Notably, a total of 66 K_hib_ proteins with 670 sites were observed in central metabolism (Table S7), and KEGG analysis showed an apparent enrichment in metabolic pathways, including glycolysis/gluconeogenesis, TCA cycle, and fatty acid degradation (**Figure 3C**). These results suggested that K_hib_ may significantly influence PC metabolism and contribute to PC progression. MG149 was identified as a highly selective inhibitor of Tip60, which was known as a writer catalyzing K_hib_ in mammalian cells (14). In order to explore the contribution of K_hib_ to PC development, the SW1990 and ASPC-1 cells were treated with MG149 to decrease the total level of K_hib_ modification. Then, we conducted the CCK8 and colony-forming assay, the results of which showed that MG149 strongly reduced the proliferation viability of the SW1990 and ASPC-1 cells compared to the control group. In addition, the results of the wounding and transwell assay suggested that MG149 suppressed the ability of migration and invasion in the SW1990 and ASPC-1 cells. The results above demonstrated that lysine 2-hydroxyisobutyrylation played an essential role in PC development and reminded us to pay more attention to 2-hydroxyisobutyrylation modification in PC. PTMs impact protein stability and function, and PTM therapies targeting proteins have made considerable advances in cancer (20, 34). It is promising that the inhibitors focusing on K_hib_ proteins may be potential targets for pancreatic cancer therapy.

Since K_hib_ modification is a new type of protein modification, it is still unexplored and lacks mature site-specific antibodies, making further in-depth studies challenging. Indeed, comparing data between cancerous and paraneoplastic tissues should be more convincing; however, given individual patient characteristics, a large number of samples are required. Based on the considerations above, we aimed to explore K_hib_ protein profiles of PC with cancer tissue and provided a new perspective for further studies. In addition, although mass spectrometry has developed rapidly, the information on low-abundance proteins and amino acid residues with low recognition sensitivity was often missed, which made subsequent studies difficult. Bao et al. developed the 2-hydr_Ensemble residue identification algorithm with artificial intelligence approaches, which was more accurate than traditional algorithms in inferring K_hib_-modified residues (35) and it could be helpful for understanding the functions and further studying the K_hib_-specific modification sites. In the current study, we first described 10367 K_hib_-modified sites on 2325 modified proteins in seven persons with pancreatic cancer and then demonstrated that K_hib_ modification played a significant role in PC development. Indeed, the potential mechanisms by which K_hib_ promoted PC progression remained unclear, more samples and studies are needed to elucidate how lysine 2-hydroxyisobutyrylation affects PC development. Our work has undeniably helped to enhance our view of this new lysine PTM. The significant amount of K_hib_ proteins identified in PC is expected to serve as an invaluable database for future pancreatic cancer-related research, providing new insights and potential targets for treating pancreatic cancer.

## Conclusions

2-hydroxyisobutyrylation at lysine is a novel type of PTMs, first reported in the histones of HeLa cells. Recently, great studies about K_hib_ modification identified in proteins have been reported in fields of diverse species. This study provides an in-depth insight into the K_hib_ protein in pancreatic carcinoma for the first time and found that the K_hib_ proteins are mainly enriched in bio-metabolic pathways. Further, we have demonstrated that K_hib_ may act as a tumor promoter in PC development and provided a new perspective to explore the multiple functions of 2-hydroxyisobutyrylation and potential treatment targets in PC.

## Materials and methods

### Patients and clinical sample collection

Seven patients with PC who had not been treated with radiotherapy were recruited for our study, and all patients independently signed written informed consent and donated tumor tissues for scientific research. This study was approved by the Tongji Hospital Research Ethics Committee and the Institutional Review Board. The enrolled patients’ demographic characteristics and clinical information are listed in Table S1. After surgery for PC, cancer tissues were rapidly dipped into MACS Tissue Storage Solution (Miltenyi Biotec GmbH, USA) and then stored at -80°C.

### Protein extraction

Samples were ground to cell powder in liquid nitrogen, and then four volumes of lysis buffer (8 M urea, 1% Protease Inhibitor Cocktail, 3 μM TSA, and 50 mM NAM) were added, followed by sonication three times on ice, after centrifugation (12,000 g at 4°C for 10 min), and collection of the supernatant. Then, the protein concentration was determined by using the BCA kit.

### Trypsin digestion

The protein solution was reduced with dithiothreitol (5 mM, 56°C, 30min) and then alkylated with 11 mM iodoacetamide (15 min, room temperature, dark conditions) and diluted with 100 mM TEAB in order to make the sample urea concentration less than 2 M. The first digestion was performed overnight at a trypsin-to-protein ratio of 1:50(trypsin: protein, weight to weight), followed by second digestion of 1:100 for 4 h.

### Affinity enrichment of lysine 2-hydroxyisobutyrylated peptides

Tryptic peptides dissolved in NETN buffer were incubated overnight with prewashed antibody beads(PTM-802, PTM Bio), followed by washing with NETN buffer and H_2_O. 0.1% trifluoroacetic acid was used to elute the bound peptides and then combined and vacuum-dried. Finally, all samples were desalted for LC-MS/MS analysis.

### LC-MS/MS analysis

Tryptic peptides were dissolved in 0.1% formic acid (solvent A) and directly loaded onto a reversed-phase analytical column (15-cm length, 75 μm i.d.). As described previously (13), the gradient comprised of solvent B at a constant flow rate of 400 nL/min on an EASY-nLC 1000 UPLC system. Then, the peptides were subjected to the NSI source, followed by tandem mass spectrometry (MS/MS) in Q ExactiveTM Plus (Thermo) coupled online to the UPLC, with the following parameter: 2.0 kV electrospray voltage, 350 to 1800 m/z scan range, and resolution of 70,000 for detection. Selected peptides MS/MS and fragments were detected in the Orbitrap at a resolution of 17,500. Depending on the data, a program of 20 MS/MS scans was carried out alternating after one MS scan with 15.0s dynamic exclusion. Automatic gain control (AGC) was programmed at 5E4. The fixed first mass was set at 100 m/z.

### Database search

The MS/MS data were manipulated using the MaxQuant search engine (v.1.5.2.8). The detailed steps have been previously described (13).

### Bioinformatics analysis

Gene Ontology annotation was performed using the UniProt-GOA database (http://www.ebi.ac.uk/GOA/). The KEGG database was applied to identify the protein pathway. The Motif-X algorithm was used to analyze the motif characteristics of the modification sites. Wolfpsort software was used to predict subcellular localization.

### Cell lines

The human normal pancreatic duct epithelial (HPDE) and human PC cell lines (ASPC-1, BXPC-3, SW1990, and Mia PaCa-2, PANC-1) cell lines used in this study were purchased from American Type Culture Collection (ATCC, Manassas, VA, USA). The cell line catalog number were shown as below: HPDE (bio-133286), ASPC-1 (bio-72969), BXPC-3 (bio-50655), SW1990 (bio-73389), Mia PaCa-2(bio-74690), PANC-1(bio-73125). HPDE, BXPC, ASPC-1, and SW1990 were cultured in RPMI 1640 medium with 10% fetal bovine serum (FBS). Mia PaCa-2 and PANC-1 were cultured in Dulbecco modified Eagle medium (DMEM) with 10% FBS. All cells were cultured in a humidified incubator at 37 °C and 5% CO2.

### Inhibitor

MG149 was used with a concentration of 74 µM, which was reported as half maximal inhibitory concentration (IC_50_) of Tip60 (27).

### Western blot

The western blotting assay was performed to detect the expression of the Khib proteins in PC tissues and cell lines. Briefly, cells or tissues were lysed by pre-formulated lysis buffer (RIPA, 1% Protease Inhibitor Cocktail, 3 μM TSA, and 50 mM NAM). The protein concentrations were measured by BCA kit (P0012S; Beyotime Biotechnology, China), and then we analyzed 50μg protein of every sample on 10% SDS-PAGE gels. All blots were visualized using ECL Kit (RM00020P; ABclonal, Wuhan, China) and the intensity of the bands was assessed by Image Lab (Bio-Rad, California, USA). The antibodies used in this study were: pan-lysine 2-hydroxyisobutyrylation antibody (1:1000; PTM-802; PTM Bio, Hangzhou, China), FITC antibody (1:50; AS011; ABclonal, Wuhan, China), β-actin antibody (1:1000; AC026; ABclonal, Wuhan, China). β-actin was used as a loading control and the intensity of the entire bands was assessed with ImageJ2 (National Institute of Mental Health, Bethesda, MD, USA). All experiments were performed three times following the same procedure.

### Immunofluorescence

Cells were spread evenly on coverslips and incubated for 24 hours, then fixed in 4% paraformaldehyde at room temperature for 15 min and permeabilized with 0.1% TX-100 for 10 min at room temperature. Then, the cells were blocked with 5% BSA in PBS for 1 hour and incubated overnight at 4°C with pan-Khib antibody (PTM-802; PTM Bio, Hangzhou, China). After washing three times with PBS, cells were incubated with the appropriate secondary antibody for 1 hour at 4°C. Coverslips were stained with DAPI and mounted. Immunofluorescence images were then observed and captured under a fluorescent microscope.

### Cell proliferation and drug cytotoxic assay *in vitro*

The viability of the SW1990 and ASPC-1 was evaluated with the cell counting kit-8 (Dojindo Laboratories Co. Ltd, Kumamoto, Japan). In brief, both SW1990 and ASPC-1 cells were seeded in 96-well plates at a density of 3 × 10^3^ cells per well. There were five replicates per group (n = 5). At the time points indicated, 10 μL of CCK-8 solution was added to each well and incubated at 37°C and 5% CO_2_ for 3 hours. Then, the absorbance at 450 nm at different time points with the plate reader (Bio-Tek Elx 800, USA) to assess cell proliferation. All experiments were performed three times following the same procedure.

### Colony formation assay

The SW1990 and ASPC-1 were seeded into 6-well plates with a density of 500 cells per well and cultured in a completed medium at 37°C and 5% CO_2_ for 14 days. Then, the indicated cells were washed with PBS, fixed with 4% paraformaldehyde, and stained with crystalline violet dye solution. After drying, the wells were photographed with a cell phone. All experiments were repeated three times.

### Wound healing assay

The indicated PC cells were cultured in 6-well plates until the cell density approached 90%; we scratched the wound vertically across the center of the well with the tip of a 200 μL pipette gently. After being washed with PBS three times, the PC cells were cultured with a serum-free medium at 37°C, 5% CO2. The result of the assay was observed and captured with a microscope (Nikon, Japan) at 0 and 24 hours, respectively. All experiments were repeated three times.

### Transwell assay

To examine cell migration capacity, the PC cells were suspended at a density of 1 × 10^5^/mL in a 24-well transwell plate (8 μm pore size; Corning) containing 200 μL of a serum-free medium in the upper transwell chamber, and 700 μL of a complete medium in the lower chamber. After co-culturing for 24 h, we fixed the PC cells on the sub-membrane surface with 4% paraformaldehyde and stained them with the crystalline violet solution. Counted the number of stained cells with a Nikon light microscope (Nikon, Japan). In the case of the invasion assay, 60 μL of Matrigel matrix gel is placed in the upper transwell chamber (BD Biosciences, America). Other operations performed were the same as cell migration assay described above. All experiments were carried out three times following the same procedure.

### Statistical analyses

One-way analysis of variance (ANOVA) and Duncan’s multiple range test were performed by SPSS v22.0. Quantitative data were analyzed by two-tailed independent Student’s t tests.

## Data availability statement

The datasets presented in this study can be found in online repositories. The names of the repository/repositories and accession number(s) can be found in the article/supplementary material. The mass spectrometry proteomics data in this study were deposited in the ProteomeXchange Consortium via the PRIDE (36) partner repository with the dataset identifier PXD029817 (http://www.ebi.ac.uk/pride).

## Ethics statement

The studies involving human participants were reviewed and approved by Tongji Hospital Research Ethics Committee and the Institutional Review Board. The patients/participants provided their written informed consent to participate in this study.

## Author contributions

YL conceived the idea and designed the study, analyzed data, and wrote the manuscript. XL, performed most of the experiment. KZ, PQ, ZD, provided help for data analysis. JW and WY supervised the entire project.

## Funding

This study was supported by the National Natural Science Foundation of China (Nos.81874062 and 82072730), and the Youth Program of National Natural Science Foundation of China (81902439).

## Acknowledgments

The study was supported by the National Natural Science Foundation of China (Nos.81874062 and 82072730), and the Youth Program of National Natural Science Foundation of China (81902439). We thank Jingjie PTM BioLab (Hangzhou, China) for technical support.

## Conflict of interest

The authors declare that the research was conducted in the absence of any commercial or financial relationships that could be construed as a potential conflict of interest.

## Publisher’s note

All claims expressed in this article are solely those of the authors and do not necessarily represent those of their affiliated organizations, or those of the publisher, the editors and the reviewers. Any product that may be evaluated in this article, or claim that may be made by its manufacturer, is not guaranteed or endorsed by the publisher.

## Abbreviations

CCK8, cell counting kit-8; K_hib,_ lysine 2-hydroxyisobutylation; K_ac,_ lysine acetylation; K_su,_ lysine succinylation; KEGG, kyoto encyclopedia of genes and genomes; LC-MS/MS, liquid chromatography with tandem mass spectrometry; OSCC, oral squamous cell carcinoma; PTM, post-translational modification; PC, pancreatic cancer; TCA cycle, tricarboxylic acid cycle; 2-HIBA, 2-hydroxybutyrate.
