# Supplementary figures and images for: Global landscape of 2-hydroxyisobutyrylation in human pancreatic cancer
Source: Front Oncol. 2022 Sep 30;12:1001807. doi: 10.3389/fonc.2022.1001807 (PMC9563853; doi:10.3389/fonc.2022.1001807)

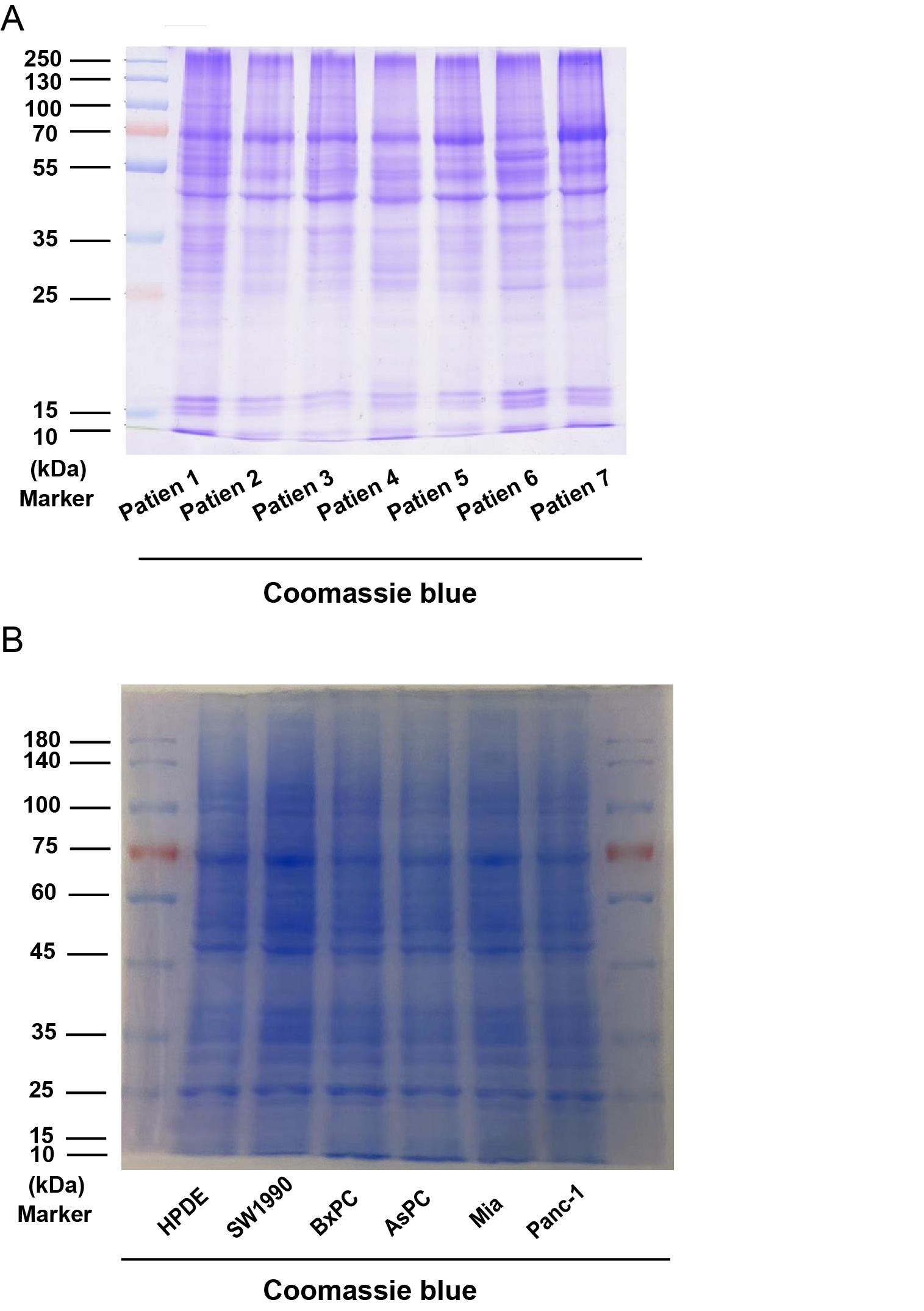

Supplement: Supplementary file 1 [file Image_1.jpeg]

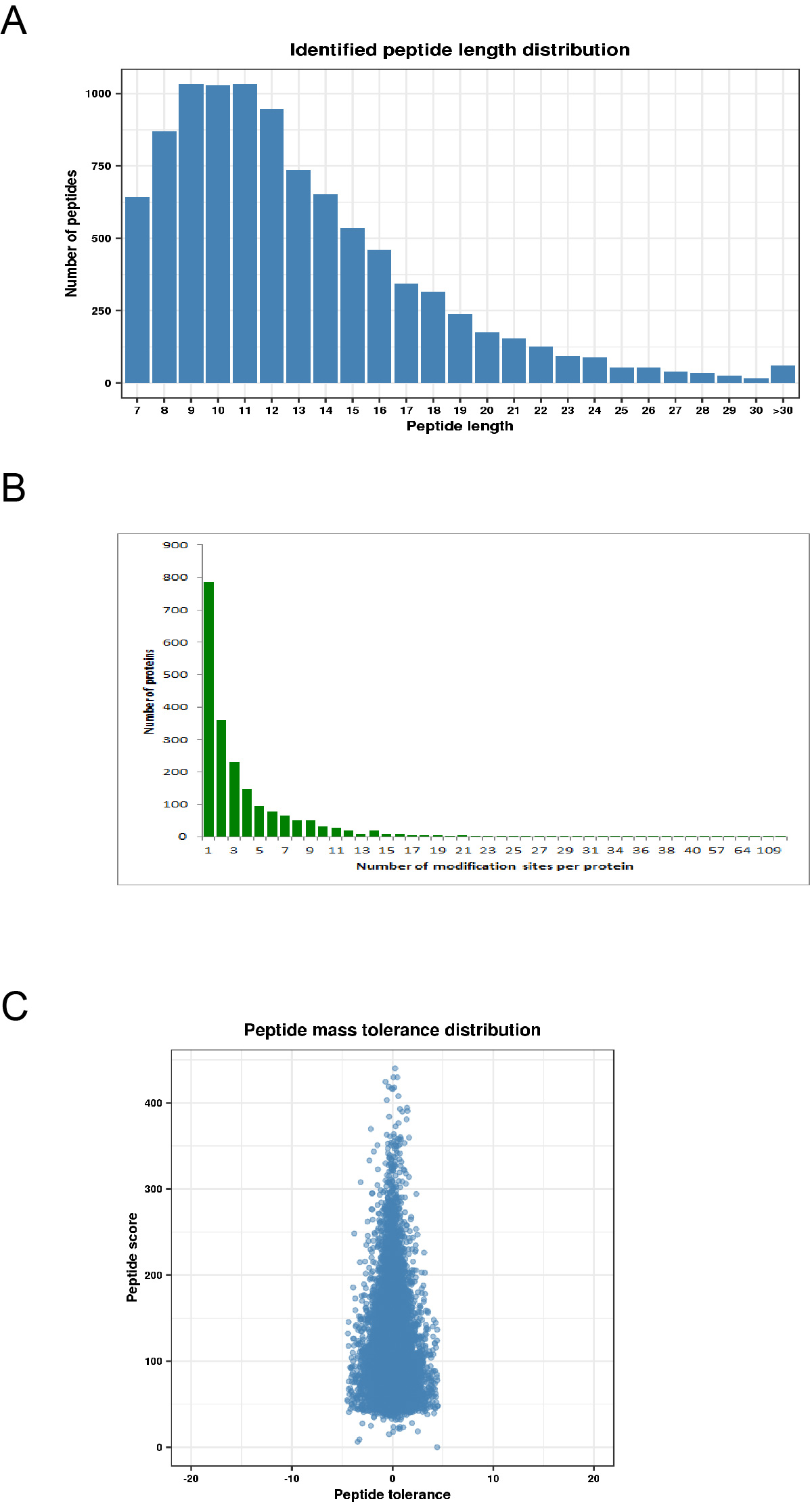

Supplement: Supplementary file 2 [file Image_2.jpeg]

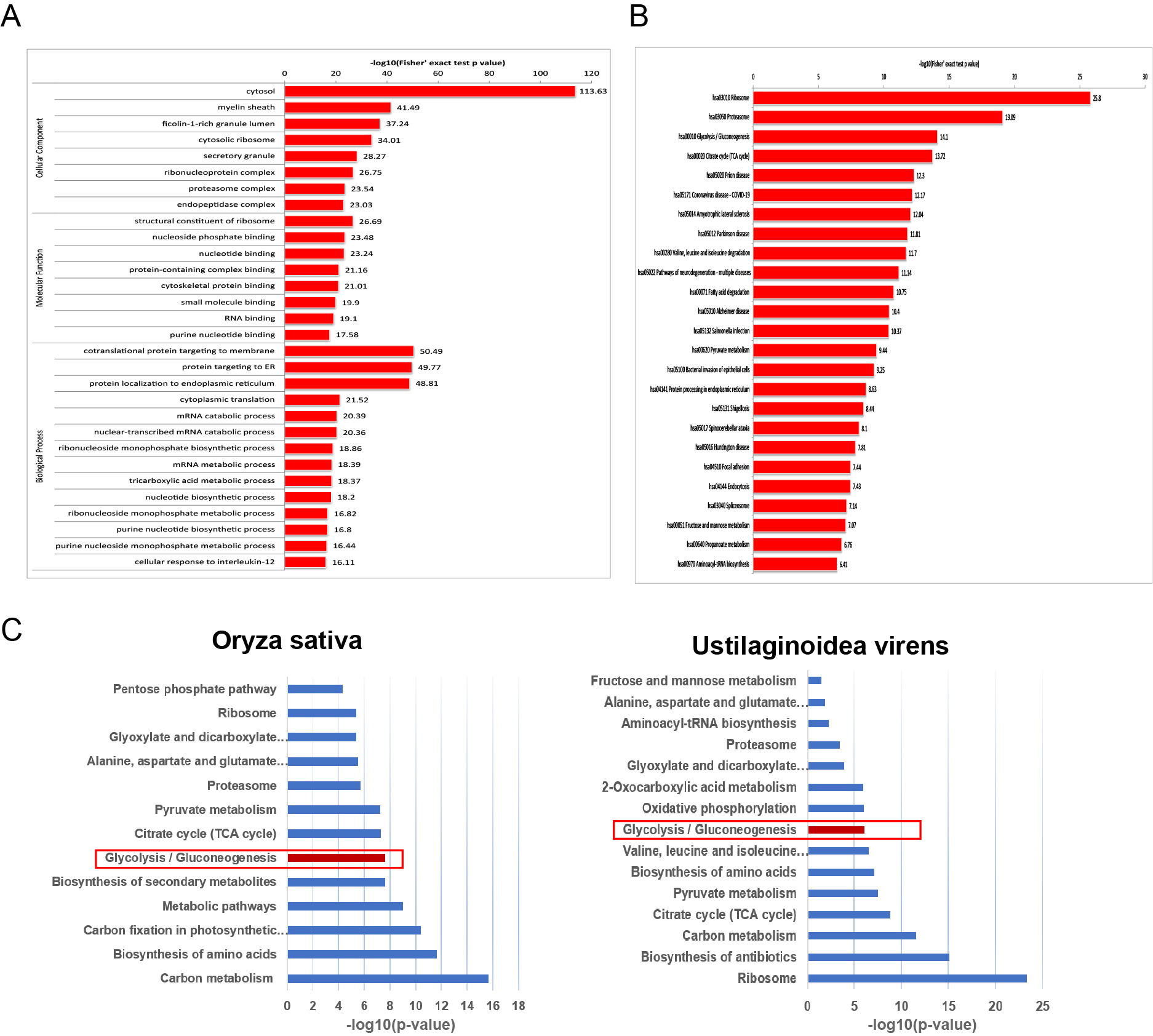

Supplement: Supplementary file 3 [file Image_3.jpeg]

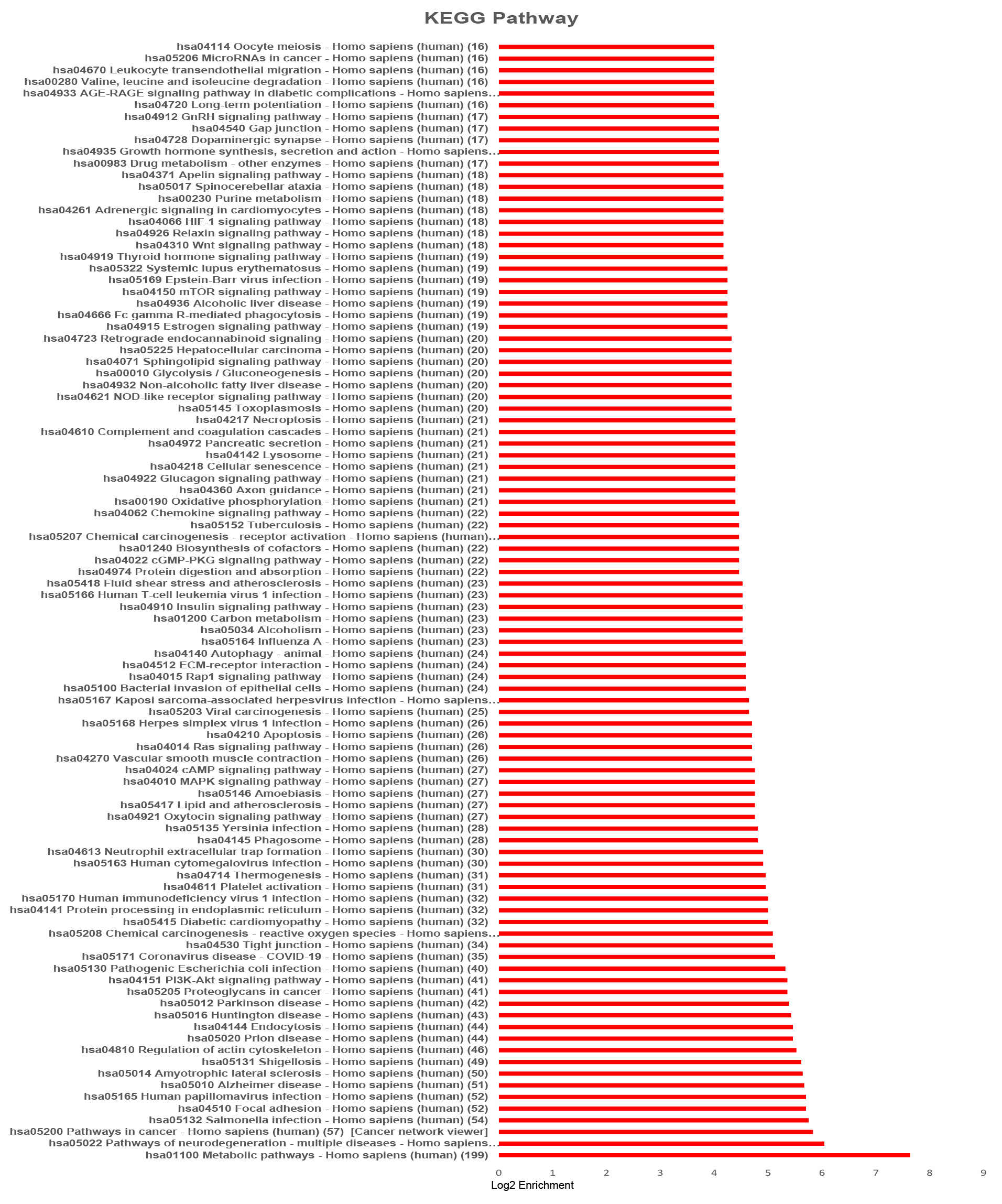

Supplement: Supplementary file 4 [file Image_4.jpeg]

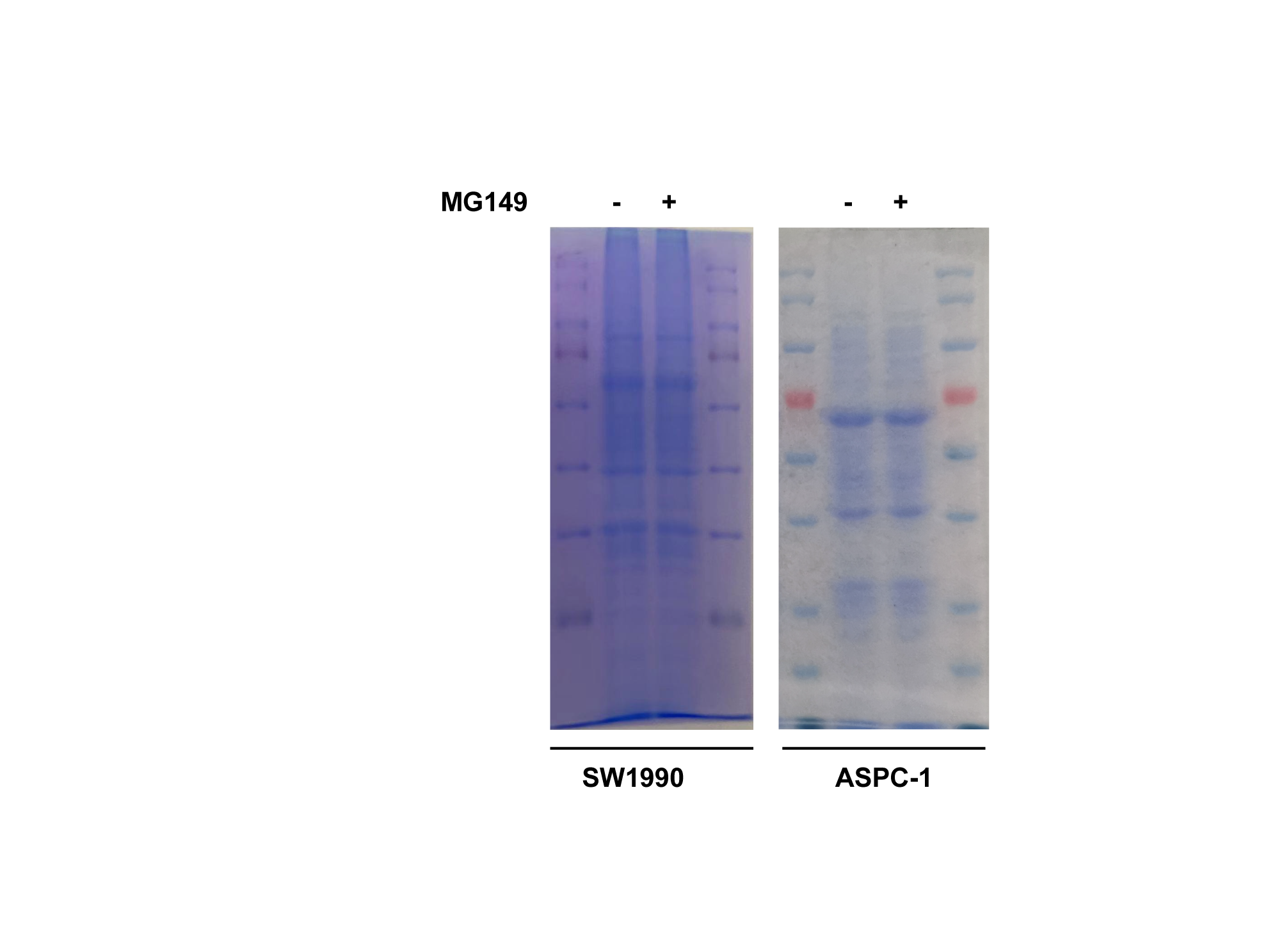

Supplement: Supplementary file 5 [file Image_5.tif]
